# Outcomes of Pediatric Patients with Excessive Postural Tachycardia After Attending an Intensive Interdisciplinary Pain Program: A Pilot Study

**DOI:** 10.3390/children12020186

**Published:** 2025-02-05

**Authors:** Cynthia Harbeck-Weber, Kelsey Klaas, Leslie Sim, Karen Weiss, April Shappell, Tracy Harrison

**Affiliations:** 1 Department of Psychiatry and Psychology, Mayo Clinic, 200 First Street SW, Rochester, MN 55905, USA; sim.leslie@mayo.edu (L.S.); weiss.karen2@mayo.edu (K.W.); shappell.april@mayo.edu (A.S.); harrison.tracy@mayo.edu (T.H.); 2 Department of Pediatrics, Mayo Clinic, 200 First Street SW, Rochester, MN 55905, USA; klaas.kelsey@mayo.edu; 3 Department of Nursing, Mayo Clinic, 200 First Street SW, Rochester, MN 55905, USA; 4 Department of Anesthesiology, Mayo Clinic, 200 First Street SW, Rochester, MN 55905, USA

**Keywords:** excessive postural tachycardia, Postural Orthostatic Tachycardia Syndrome (POTS), intensive interdisciplinary pain treatment, rehabilitation

## Abstract

Background/Objectives: Adolescents with autonomic disorders who attend Intensive Interdisciplinary Pain Treatment (IIPT) programs report improvements in functioning. However, it is unclear whether they experience corresponding improvements in physiological measures. As such, the aim of this pilot study was to examine changes in physiological measures in youth attending an IIPT program who demonstrated excessive postural tachycardia on an active stand test. The secondary goal was to examine associations between physiological measurements and self-reported measures of chronic orthostatic intolerance (cOI) and functioning. Methods: At admission and discharge, eighteen adolescents and young adults (AYAs) attending IIPT (*M* age = 17.39 years; SD = 2.15 years) completed an active stand test, measures of breathing rate and muscle tension, as well as self-reported measures of cOI symptoms and functioning. Results: AYAs showed significant reduction in active stand test heart rate increase (*p* < 0.001; *d* = 1.07) and maximum heart rate (*p* = 0.002, *d* = 0.76) from admission to discharge. Improvements were also observed in resting respiration rate (*p* = 0.001, *d* = 89) and resting trapezoid tension (*p* = 0.03, *d* = 0.49). Although patients showed significant improvements on self-report measures of functioning (*p* < 0.001, *d* = 1.78), changes on subjective report of cOI symptoms did not reach significance. Exploratory analyses that only included patients with a POTS diagnosis were consistent with the overall results. Conclusions: Youth who demonstrated excessive postural tachycardia on active stand test at admission to an IIPT showed significant improvements from admission to discharge in their active stand maximum heart rate and heart rate increase, as well as respiration rate, muscle tension, and reports of their functioning. Future research is necessary to examine the mechanisms of change that contribute to symptom improvement.

## 1. Introduction

Excessive postural tachycardia is one of the diagnostic criteria for Postural Orthostatic Tachycardia Syndrome (POTS). A diagnosis of POTS in individuals 12 to 18 years of age consists of at least three months of prominent, bothersome symptoms associated with moving from a supine to standing position and, with head up tilt table testing, an increase in heart rate of ≥40 beats per minute without associated orthostatic hypotension (>20/10 mm Hg drop in blood pressure). For adolescents and young adults 19 and older, a heart rate increase of >+30 bpm during tilt table testing is required [1].

POTS affects up to 1% of adolescents and is one of the autonomic nervous system disorders characterized by chronic positional symptoms along with specific heart rate changes. It is associated with a heterogeneous constellation of symptoms ranging in severity from mild to disabling and frequently includes dizziness, syncope, blurred vision, fatigue, weakness, gastrointestinal dysfunction, headaches, abdominal pain, musculoskeletal pain, tremors, and brain fog [1,2]. Not surprisingly, these symptoms can contribute to impairments in functioning [3]. Youth with POTS report difficulties with self-care (hygiene, eating, sleeping, and exercise), as well as inconsistencies in daily routines, school attendance, homework completion, extra-curricular activities, and socializing with friends [4]. Some research suggests that the symptoms of pediatric patients with POTS improve over time [5]. However, symptoms of POTS often last into adulthood [6] and improvements may take years, even with conventional treatments such as increasing fluid and salt intake [6,7]. Even five years after diagnosis, most young people with POTS continue to experience symptoms, with fatigue, dizziness or lightheadedness, and tachycardia most frequently reported [7].

As patients with POTS report significant distress over their symptoms [8], effective interventions are needed to decrease the course and severity of symptoms. Current interventions include both non-pharmacologic and pharmacologic interventions. Medication options include off label use of agents that attempt to address problematic symptoms or underlying mechanisms of POTS, such as medications that lower heart rate or expand intravascular volume [3]. Because no single medication or medication class has been found to be helpful in a majority of patients, current research is attempting to match medication recommendations to individual baseline measurements (e.g., urinary sodium, heart rate variability) that implicate specific causal factors [9]. Lifestyle changes such as increasing sodium and fluids, as well as engagement in regular physical activity, are associated with symptomatic improvement. When symptoms are severe and disabling, intensive interdisciplinary treatment programs (IITPs) may be indicated. IITPs consist of inpatient or day treatment programs that include at least three disciplines (typically physician, psychologist, and physical therapist) and focus on functional restoration [10]. Most programs include full days of treatment and last for three to four weeks. Research suggests pediatric patients with POTS attending IITPs show significant improvements in improved overall functioning, decreased psychological distress including depression, decreased pain intensity and pain catastrophizing, and improvement in individualized functioning goals [4,11,12].

To examine improvements in pediatric patients with POTS attending an IIPT, research to date has relied on self-report measures, including measures of symptoms attributable to autonomic dysfunction (e.g., COMPASS 31), age-appropriate functioning (e.g., Functional Disability Inventory), quality of life (e.g., Symptom Checklist 90), and performance in meeting individualized goals (e.g., Canadian Occupational Performance Measure). While research shows substantial improvement from IIPT participation across all domains of self-report measures, it is unclear whether adolescents experience corresponding improvements on objectively measured features of orthostatic intolerance.

Currently, the only validated tool for diagnosis of POTS in adolescents is the head up tilt table testing [13]. During head-up tilt table testing, patients are secured to a table with a footplate in a supine position for at least 10 min, and then the table is elevated to approximately 70 degrees (at least 60 degrees) for a minimum of 10 additional minutes. Although this test provides a reliable measurement of hemodynamic profiles associated with orthostatic intolerance, there are several disadvantages of this test for measuring outcomes. These tests are resource intensive and costly, requiring specialized equipment, space, and personnel. In addition, many patients may be hesitant to complete testing, especially repeat testing, due to associated symptom exacerbations that often occur. Due to patient discomfort and required resources, the head up tilt table test is not always available or recommended. Therefore, a less resource intensive and better tolerated procedure, an active stand test, is sometimes used in clinical practice. An active stand test requires minimal equipment and is not dependent on specialized staff members and space. The assessment can be completed in a typical hospital or clinic exam room by trained staff members (e.g., medical assistant, nursing staff members) as opposed to trained technicians that are required for the head up tilt table test. While not validated for diagnosis of POTS in adolescents, it has been validated in adults [14].

During an active stand test, the patient remains supine for at least 5 min and then stands unassisted for 10 min [15]. A blood pressure cuff is utilized to obtain heart rate and blood pressure measurements at multiple time points during the 10 min. Although few studies have compared results from head up tilt table and active stand test due to differences in test conditions, these tests would not be expected to produce exactly the same results. In contrast to the head up tilt table test, in which patients are supported by the exam table, patients completing the active stand test contract leg muscles as they stand, which compress the lower limb veins, forcing venous return to the heart, potentially mitigating the tachycardic response and associated symptoms of palpitations, lightheadedness, and presyncope. In fact, the head up tilt table test produces significantly larger heart rate changes than the active stand test in adults [14]. However, in a setting without head up tilt table test equipment, the active stand test can be used to assist with diagnoses and monitor improvements in orthostatic vital signs [16].

Although limited research exists on adolescents and young adults with POTS, even less is known about patients who experience chronic orthostatic intolerance (cOI) with associated functional impairment but do not demonstrate the excessive postural tachycardia needed for a diagnosis of POTS, or patients with excessive postural tachycardia (without known causes such as deconditioning, anorexia nervosa) but do not fulfill diagnostic criteria for POTS. Therefore, the current study sought to include all patients admitted to an IIPT who met criteria for excessive postural tachycardia. The primary aim of this study was to examine physiological changes following IIPT program participation for adolescent and young adult patients demonstrating excessive postural tachycardia at admission. Specifically, we hypothesized that young people demonstrating excessive postural tachycardia during the active stand test would demonstrate significant improvement in their standing orthostatic heart rate change and maximum heart rate, as well as their resting respiration rate and muscle tension, after completing an intensive treatment program. Second, we hypothesized that improvement in positional heart rate change would be associated with improvements in other physiological measurements as well as self-report measures of autonomic symptoms and age-appropriate functioning. Finally, exploratory analyses were conducted to determine the outcomes on the subset of patients with a formal POTS diagnosis.

## 2. Methods

### 2.1. Participants

Participants were identified from 140 young people consecutively admitted to an IIPT program in the Midwest who completed an active stand test at admission and discharge. Patients who met criteria for excessive heart rate increase during active stand test at admission were included in the study. An increased heart rate was defined as >30 beats per minute (bpm) for individuals 19 and older and ≥40 bpm for youth under 19. Inclusion criteria for the pain program included (1) pain and/or other symptoms lasting longer than 3 months and severe enough to interfere with age-appropriate functioning, (2) aged 12–22 years, (3) English-speaking, (4) able to complete self-care (e.g., toileting, eating) independently, (5) having a parent attend the complete program along with the patient, and (6) being able to commit to a program focused on functional restoration rather than diagnostic evaluation or symptom management. Exclusion criteria included a diagnosis of anorexia nervosa or bulimia nervosa, or having a substance abuse concern or a mental health concern (e.g., PTSD) severe enough to interfere with treatment. Patients unable to eat or toilet independently were also excluded. All patients completed the program. One patient chose not to participate in biofeedback due to lack of insurance reimbursement. As one patient was completing the program for a second time, they did not participate in the self-report component of the research, thus those data are not available. One additional patient did not complete self-report measures at discharge.

### 2.2. Measures

During the active stand test, patients were instructed to lay down for 5 min on a flat surface and then were directed to stand and maintain an upright position for the next 10 min. The patient and the providers were instructed not to interact during the 10-min period. Patients kept their arms next to their torso when supine and standing. Nursing staff members measured heart rate and blood pressure after laying supine for 5 min and at 1, 5, and 10 min after standing using an automated oscillometric sphygmomanometer. Patients were instructed to rise from the supine to the standing position without assistance. All patients in the study were able to rise and stand without the provision of physical support.

Resting respiration (RR) rates were measured using an adjustable belt with a respiratory sensor placed approximately one inch above patients’ umbilicus to measure the pace of breathing. The belt was attached to a computer using Thought Technology hardware and BioGraph Infiniti software (version 6.8) (both from Thought Technology, Quebec, QC, Canada). Biofeedback therapists encouraged patients to breathe normally for 5 min during the admission session and to breathe using their best skills for 5 min at the final session. Biofeedback therapists recorded average respirations per minute. Although optimal rates of relaxed diaphragmatic breathing for adults are often considered to be approximately six breaths per minute [17], recommended breathing rates for physiologic relaxation in children are not well established [18] and it can be difficult for adolescents to achieve rates below 8 bpm [19]. Therefore, a goal of 6–8 breaths per minute was established.

Bilateral shoulder tension was measured by surface electromyography (sEMG), with an electrode placed on the left and right trapezius muscle using Thought Technology hardware with BioGraph Infiniti software. Muscles are generally considered to be relaxed when they measure below 2 μV, accounting for electrical activity from action potentials within muscles. The baseline recording session lasted 5 min and the average sEMG rating for both the left and right trapezius was recorded in the patient’s chart.

The POTS Symptom Summary is a 10-item self-report questionnaire developed by the authors to assess symptoms related to autonomic dysfunction, including rapid or increased heart rate, nausea/vomiting, dizziness/swimming sensation, blurred vision, feelings of weakness, feeling shaky, turning pale, clammy skin, or presumed syncope. The first nine items are rated on a 1–4 scale ranging from “have not had” to “severe”. The last item, “Have you ever completely lost consciousness after a spell of dizziness?”, is rated yes/no. Scores are summed for a total score ranging from 0–38. When separately analyzed, Cronbach’s alpha for the scale was 0.89 at admission and 0.91 at discharge, suggesting excellent internal consistency.

The Functional Disability Inventory (FDI) [20] is a 15-item questionnaire that asks respondents to rate their difficulty in completing age-appropriate home, school, and social tasks on a 5-point Likert scale. Responses range from 0 (no trouble) to 4 (impossible). Total scores range from 0–60, with four levels of disability: no–minimal (0–12), mild (13–20), moderate (21–29), and severe (>29). Multiple studies have found the FDI to have good reliability and validity [21,22].

### 2.3. Procedure

Patients completed a battery of standardized, clinical self-report measures on day 1 or 2 of their IIPT program, typically before any treatment had begun, and again on day 16 or 17 of their program. Study data were collected and managed using REDCap (Research Electronic Data Capture) electronic data capture tools hosted at Mayo Clinic [23,24]. REDCap is a secure, web-based software platform designed to support data capture for research studies, providing (1) an intuitive interface for validated data capture; (2) audit trails for tracking data manipulation and export procedures; (3) automated export procedures for seamless data downloads to common statistical packages; and (4) procedures for data integration and interoperability with external sources. Nurse case managers administered the active stand test, and biofeedback therapists recorded surface EMG and respiration rates during a 5-min baseline recording conducted at the beginning of each biofeedback session. Patients also completed a blood assay during their admission process. Information from charts was abstracted by the first and last author. An expert in pediatric POTS (KK) reviewed electronic medical records to determine POTS diagnosis. Data were analyzed using SPSS v25. This retrospective chart review study was determined to be exempt by the Mayo Clinic Institutional Review Board.

### 2.4. Setting

Patients participated in a 3-week (15 days treatment plus 2 admission days) group-based IIPT program that focuses on functional restoration for patients with chronic pain and other symptoms (e.g., fatigue, dizziness, nausea), including symptoms of autonomic dysfunction and POTS. The length of the program is similar to other IIPT programs [10,25]. Patients attended the program from 8 am to 5 pm daily and returned to their hotel or other local lodging for the evening and weekends, where they were encouraged to continue practicing program concepts such as maintaining a structured schedule and relaxation. They received daily physical therapy, occupational therapy, relaxation training, cognitive-behavioral therapy, and recreation therapy for a total of approximately 120 h of intervention. The overall orientation of the program is biopsychosocial, with interventions aimed to optimize medication use and improve sleep, eating, drinking fluids, and movement (bio), cognitive-behavioral interventions such as psychoeducation, reduced focus on symptoms, and stress management (cognitive), as well as social activities and return to school/leisure/hobbies (social). Parents/caregivers were an integral part of the program and received over 33 h of intervention, including parent groups, family groups, and meetings with the patient’s nurse case manager. Although the overall goal of the program is for patients to return to full age-appropriate living when they finish the program, nurse case managers and other providers helped patients identify their own individual goals and steps to reach those goals. The functional restoration goals of the program typically included full time, in person school attendance with on-time schoolwork completion and full participation in family life (e.g., chores, family activities) and extracurricular activities.

Group topics of the program are wide-ranging and include pain and symptom management, general wellness (e.g., sleep hygiene, normal eating), stress management and mental health (e.g., coping with stress, perfectionism, anxiety, depression), tools for school success, and other skills to improve success at returning to age-appropriate activities. Throughout the program, parents and/or caregivers are encouraged to decrease attention to their child’s symptoms and to use behavioral parenting strategies to motivate engagement in typical expectations for their youth.

### 2.5. Statistical Analyses

Dependent samples *t*-tests were used to compare parameters from admission and discharge active stand test and self-report measures. Due to multiple comparisons, Bonferroni corrections were utilized for each group of tests (physiological and self-report measures). For physiological measures, *p* value significance was established at *p* < 0.0125. For self-report measures, a Bonferroni correction of *p* < 0.025 was utilized to determine significance. Effect sizes were calculated using Cohen’s *d*. Next, correlations examined the relationships between changes in heart rate increase, maximum heart rate, resting respirations, trapezoid muscle tension, change in self-reported symptoms, and change in self-reported functioning. Finally, exploratory analyses using dependent samples *t*-tests examined changes between admission and discharge for patients with POTS.

## 3. Results

The study (see Table 1) included 18 patients aged 13–21 (*M* = 17.39; *SD* = 2.15). More than half of the patients were female (61%) and all patients self-identified as White (100%). Patients resided in 13 different states, representing all 5 major geographical areas of the United States. Nine patients met criteria for POTS, including >3 months of orthostatic intolerance and positive head up tilt test (excessive postural tachycardia for age in the absence of orthostatic hypotension). Two patients had been diagnosed with POTS at a different institution. Five additional patients reported chronic orthostatic intolerance, and a head up tilt table test was not completed or results were not available. Two patients had chronic pain without reported orthostatic intolerance symptoms. Patients typically reported chronic pain in multiple locations, ranging from moderate to severe (see Table 1). Admission testing revealed that a majority (*n* = 16; 89%) of the patients had low levels of Vitamin D and a minority (*n* = 3; 17%) had low levels of Ferritin.

### 3.1. Medication Use

At admission, 56% of patients (*n* = 10) were taking medications or supplements for their autonomic disorder symptoms, including fludrocortisone, midodrine, metoprolol, DDAVP, and salt supplements (Table 2). Many patients (22–33%) were also taking antidepressants, pain medications, and antiemetics. A smaller number of patients (1–3, 6–17%) reported taking sleep medications, stimulants, narcotics, benzodiazepines, and muscle relaxants.

Throughout the intervention, the majority of patients continued on the medications they were previously prescribed. One patient was started on fluoxetine toward the end of the program after meeting with a psychiatrist and one patient began using salt supplementation. Patients were given recommendations for vitamin D supplementation as needed. Eight (44%) of the patients tapered or stopped some of their medications, including zolpidem, amitriptyline, clonidine, duloxetine, fludrocortisone, lorazepam, midodrine, ondansetron, ramelteon, topiramate, tramadol, acetaminophen, and various supplements.

### 3.2. Changes in Physiological Measures Between Admission and Discharge

Dependent samples *t*-tests on parameters from the active stand test found a significant decrease from admission to discharge in the participants’ heart rate change upon standing, *t*(17) = 4.57, *p* < 0.001. Similarly, maximum heart rate during the active stand test decreased significantly from admission to discharge, *t*(17) = 3.24, *p* = 0.005. For biofeedback measures, the average number of respirations significantly decreased from admission to discharge, *t*(16) = 3.68, *p* = 0.001. Trapezoid tension trended toward a significant decrease from admission to discharge, *t*(16) = 2.03, *p* = 0.030. Medium to large effect sizes were found on all physiological measures (Cohen’s *d* ranges from 0.47–1.08; see Table 3).

At discharge, 15 (83%) individuals no longer displayed excessive postural tachycardia on active stand test, compared with 0 at admission. Additionally, five more patients demonstrated respiration rates within the goal rate (6–8) at discharge than at admission. Similarly, five more patients demonstrated relaxed muscles at discharge than admission. All patients who changed from one category to another changed in the direction of clinical improvement.

### 3.3. Changes in Self-Reports of Symptoms and Functioning Between Admission and Discharge

Individuals reported significant improvements in scores on the FDI from admission to discharge, *t*(15) = 7.14, *p* < 0.001, associated with a large effect size (*d* = 1.78; see Table 3). Participants’ scores on the POTS symptoms measure trended toward a significant decrease from admission to discharge, *t*(15) = 2.08, *p* = 0.028, with a moderate effect size (*d* = 0.52).

### 3.4. Relationship Between Changes in Physiological Measures and Self-Report Measures

Change in heart rate increase between admission and discharge was not significantly correlated with change in maximum heart rate *r*(18) = 0.124, *p* = 0.62, change in resting respirations *r*(17) = −0.28, *p* = 0.27, change in trapezoid muscle tension *r*(17) = −0.10, *p* = 0.70), change in self-reported symptoms *r*(16) = 0.48, *p* = 0.06, or change in self-reported functioning *r*(16) = 0.29, *p* = 0.28.

### 3.5. Outcomes for Patients with a POTS Diagnosis

As these are exploratory analyses, a Bonferroni correction was not utilized. Dependent samples *t*-tests on parameters from the active stand test found a significant decrease from admission to discharge in patients with POTS’ heart rate change upon standing, *t*(10) = 3.50, *p* = 0.006. Similarly, maximum heart rate during the active stand test decreased significantly from admission to discharge, *t*(10) = 2.85, *p* = 0.009. The average number of respirations significantly decreased from admission to discharge, *t*(9) = 4.30, *p* = 0.002. Trapezoid tension did not show a significant difference between admission and discharge, *t*(9) = 1.05, *p* = 0.16. From admission to discharge, individuals with POTS reported significant improvements in functioning, *t*(8) = 5.03, *p* < 0.001, and POTS symptoms, *t*(8) = 2.77, *p* = 0.012.

## 4. Discussion

This pilot study is the first to document significant physiological improvements in patients demonstrating excessive postural tachycardia following participation in a 3-week IIPT. Patients experienced large improvements in their supine to standing heart rate change, supine to standing maximum heart rate, and resting respirations. Moderate improvements were seen in their resting trapezoid muscle tension. Given the short time frame of measurement, the significant progress in physiological change is noteworthy. The findings were consistent in the subset of patients meeting diagnostic criteria for POTS, with the exception that patients with POTS additionally reported a significant improvement in their OI symptoms.

Most patients reported cOI, with half of the patients meeting criteria for POTS. POTS is a heterogenous condition with uncertain underlying pathophysiologic mechanisms. Proposed mechanisms include partial autonomic neuropathy, persistent tendency toward hypovolemia, and central hyperadrenergic state, with many patients having multiple pathophysiologic contributors [26]. Thus, a comprehensive program that targets multiple physiological mechanisms may be most effective. Low muscle tone, small calf size, and general deconditioning may lead to decreased skeletal muscle pump activity, which then provides insufficient venous return to the heart [3,13,27]. Based on this mechanism, it is possible that the daily physical therapy program and increased functional activity contributed to the improvements seen in physiological measures of orthostatic intolerance. Exercises designed for adolescents and young adults were emphasized, including both cardiac activity (e.g., walking, running, playing sports, dance) and strength training with both free weights and a weight machine. Other movement activities were also built into the day to increase overall fitness, including walks, brief periods to play sports, yoga, and muscle stretching.

To address the low blood volume that is commonly seen in patients with chronic OI, increased fluid and salt is commonly recommended [3,13,15]. Patients in this IIPT were encouraged to drink sufficient fluid to produce clear or pale yellow urine and eat salty food and snacks. They were also supported to eat three meals plus two snacks per day and eliminate food avoidance due to symptoms. One patient started salt supplements, and several patients tapered off salt supplements and relied on eating to obtain their sodium. As such, it is possible that patients’ regular dietary intake, as well as increases in salt and fluid consumption, contributed to their improvement. Because most patients continued or decreased their prescribed medications, medication changes are not likely to account for physiological improvements. In fact, patients who reduced their medications also experienced significant improvements in physiological measures.

In addition to improvements in physiological measures of orthostatic symptoms, patients also demonstrated significant improvements on other physiological parameters, such as resting respirations and muscle tension. During the program, biofeedback providers helped patients learn to recognize and reduce their muscle tension as well as to control their breathing and lower their breathing rate to a goal of 8–10 breaths per minute. Daily opportunities for additional relaxation practice were also provided. This is likely an essential component of the program, as reduced sympathetic arousal may contribute to reduced CNS inflammation, which may impact orthostatic intolerance. The autonomic nervous system is likely to play a fundamental role in the development and maintenance of orthostatic intolerance and POTS [28] and more research is necessary to examine physiological correlates of hypothalamic-adrenal-axis (HPA) dysfunction.

Finally, symptoms of anxiety and depression are often comorbid in pediatric patients with orthostatic intolerance and/or POTS [11,12,29] and frequently contribute to symptom burden and functional impairment, as well as having an impact on the HPA axis [30]. In this IIPT program, patients received over 30 h of cognitive-behavior oriented psychoeducation and group therapy, with many of the sessions targeted at reducing symptom catastrophizing, anxiety, and depressed mood. Behavioral activation (a known treatment for depression) was also included as patients were supported to remain active all day and engage in daily activities designed to promote socialization and fun. Prior research has shown cognitive behavior therapy interventions promote biological changes in patients with various psychiatric conditions [31]. As such, these psychological interventions may have also contributed to the physiological improvements in this sample.

Contrary to our hypothesis, the pre-post intervention changes in orthostatic and maximum heart rate were not related to patients’ subjective report of their symptoms or functioning. This finding is consistent with earlier research demonstrating that patients with a 30–39 bpm change upon standing did not report significantly lower symptoms than those with a >40 bpm heart rate change upon standing [32]. Until more is learned about the relationship between subjective and objective measures of orthostatic intolerance in the pediatric population, it will be important to include both subjective and objective measures to assess outcome. Future research should also examine whether showing patients their improvements in physiological measures will encourage their ongoing engagement in physical activity and other therapeutic strategies following dismissal, which could also lead to further symptomatic improvement.

This study has several limitations, including the small and racially homogeneous sample. In addition, the patients in this sample were enrolled in the study due to their excessive postural tachycardia but were not all formally assessed for or diagnosed with POTS. Future research should carefully document participants’ symptoms, physiological measures, and provide diagnostic certainty.

Another limitation to consider represents the severity of the sample. Because most of the patients were experiencing major functional impairments (i.e., unable to attend school, participate in age-appropriate activities), the findings of the study may not generalize to populations of youth with chronic orthostatic intolerance who are less functionally disabled. In addition, since the orthostatics were measured at admission and discharge from the program, it is unclear whether the improvements would be sustained in the long term. Finally, the timing of the orthostatic measurements was not standardized; given the circadian fluctuations of the autonomic nervous system, future research should standardize the timing of these measurements. HR and blood pressure were measured intermittently during the active stand test, and future research may wish to consider continuous measurement.

Future research using larger, more diverse samples is necessary to clarify the generalizability of these findings. Studies with advanced methodological rigor, including either a waitlist control or randomized control group, as well as follow-up data, would also increase our confidence in these data. Additional research examining specific components within the IIPT (physical movement, increased salt, fluids, cognitive-behavioral therapy, increased social interactions) will be helpful to determine which components have the strongest impact on improved symptoms and functioning. This information could be used to streamline or even shorten the treatment length for patients with POTS. Finally, potential adverse effects could be investigated with qualitative research specifically asking patients about their experience with IIPT.

Despite the need for additional research, the current findings offer significant encouragement to pediatric patients experiencing chronic orthostatic intolerance or POTS. Attending an intensive but relatively brief (3 week) program contributed to physiological improvements in their response to standing, resting respiration rate, and resting muscle tension. Additionally, patients reported significant improvements in their functioning; together these impacts may translate into decreased disease burden and improved quality of life.

## Figures and Tables

**Table 1:** Demographic and symptom characteristics at admission.

Characteristic	*n*	%	M	SD	Range
Sex at birth—Female	11	61			
Age			17.39	2.15	(13–21)
Formal POTS diagnosis	11	61			
Pain Type					
Headache	10	56			
Abdominal Pain	9	50			
Musculoskeletal pain	7	39			
Other pain	3	17			
Pain Severity (1–10 scale)			6.61	1.79	(4–10)
Pain Duration (in months)			35.95	32.53	(3.6–108)
Low Vitamin D	16	89			
Low Ferritin	3	17			

**Table 2:** Medications at admission.

	Admission	
Patients Receiving This Medication Class	*n*	%
Medications commonly		
prescribed for autonomic disorder	10	56
Antidepressants	6	33
Pain medications	5	28
Antiemetics	4	22
Sleep medications	3	17
Stimulants	2	11
Benzodiazepines	2	11
Muscle relaxants	2	11
Anticonvulsants	1	6
Opioids	1	6

**Table 3:** Comparison of Admission and Discharge Autonomic Nervous System and Self-report Measures.

Measure	Admission		Discharge		*t*(df)	*p*	Cohen’s *d*
	*M*	*SD*	*M*	*SD*			
HR increase	44.72	12.78	26.44	9.67	4.569 (17)	<0.001	1.07
Maximum HR	117.83	15.08	103.33	17.72	3.24 (17)	0.002	0.76
Resting Respirations	13.12	4.04	9.84	4.38	3.69 (16)	0.001	0.89
Trapezoid tension	4.05	5.09	1.76	1.00	2.03 (16)	0.03	0.49
POTS Symptom summary	20.56	7.07	18.50	7.00	2.07 (15)	0.04	0.52
FDI	27.13	12.27	11.63	9.93	7.149 (15)	<0.001	1.78

Note: Trapezoid tension is an average of the right and left trapezoid muscle tension; POTS Symptom Summary is a summary of patient’s self-report of autonomic disorder symptoms (range = 0–38); FDI = Functional Disability Inventory (range = 0 to 60).

## Data Availability

The data presented in this article are not readily available because they are part of an ongoing study. Requests to access the database should be directed to the first author.
